# Radiated and guided optical waves of a magnetic dipole-nanofiber system

**DOI:** 10.1038/s41598-018-38115-z

**Published:** 2019-03-05

**Authors:** Shaghik Atakaramians, Feng Q. Dong, Tanya M. Monro, Shahraam Afshar V.

**Affiliations:** 1 School of Electrical Engineering and Telecommunications, UNSW Sydney, Sydney, NSW 2052 Australia; 2 Institute of Photonics and Optical Science, School of Physics, The University of Sydney, Sydney, NSW 2006 Australia; 3 Laser Physics and Photonic Devices Laboratories, School of Engineering, University of South Australia, Mawson Lakes, SA 5095 Australia

## Abstract

Nanophotonics–photonic structures with subwavelength features–allow accessing high intensity and localized electromagnetic field and hence is an ideal platform for investigating and exploiting strong lightmatter interaction. In particular, such a strong light-matter interaction requires investigating the interaction of a magnetic dipole with the electromagnetic field– a less-explored topic, which has usually been ignored within the framework of electric dipole approximation. Motivated by recent advances in the emerging field of multipolar nanophotonics, here we develop an analytical model that provides a new insight into analyzing a magnetic dipole and a nanofiber. This method enables us to examine the effect of second term in the multipolar expansion of light-matter interaction, *magnetic dipole approximation*, with individual guided and radiation modes of the nanofiber. This is a critical key in developing nanophotonic integrated devices based on magnetic nature of light for super-imaging, biosensing, and optical computing.

## Introduction

Nanophotonics–photonic structures with subwavelength features–create unique opportunity for subwavelength control of light. Light-matter interaction–within the confined and subwavelength region of electromagnetic (EM) fields–has been explored (still being explored) within the framework of electric dipole (ED) approximation for linear and nonlinear optical processes^1–7^ and has been shown to challenge conventional theories in these processes and led to unexpected experimental results^5,7,8^. In addition, two emerging fields of dielectric metadevices^9^ and quantum optics^10^ have also been greatly affected by the strong nano-scale confined light-matter interaction within the framework of ED approximation.

In the context of metadevcies, it has been shown that dielectric nanoparticles with high refractive indices can be used as the fundamental block in metadevices due to their strong magnetic responses^11–24^. The Mie scattering of a plane-wave from high-index dielectric nanoparticles shows series of alternative magnetic and electric resonances with an enhanced and dominant MD response. Such an enhanced magnetic response can also be achieved when an ED is placed in vicinity of a high index nanofiber^25^ and nanospheres^9^. In the content of quantum optics, the interaction of quantum emitters with subwavelength waveguides has led to an emerging research field of Chiral Quantum Optics^10^, where the guided modes show strong spin-orbit angular momentum depending on the direction of the propagation. Quantum emitters with circular dipole moments can couple differently into forward and backward guided modes of a subwavelength waveguides opening avenues for development of single photon isolators, circulators and switches^10,26–29^.

A common missing element in nanophotonics examples discussed above is the investigation of light-matter interaction beyond the ED approximation, i.e. examining the effect of second term in the multipolar expansion of light-matter interaction, *magnetic dipole approximation*^30–33^. Recently, there is a great interest to understand the effect of magnetic dipole approximation for the following main reasons: (1)- atomic or molecular energy-level transitions with dominant magnetic dipole nature^34–36^, (2)- possibility to selectively excite the MD transitions^31,37^ (3)- possibility to selectively access dominant magnetic or electric responses in high index optical nanostructures^9,25^ and (4)- the existence of confined and enhanced EM fields with strong spin-orbit angular momentum in nanophotonic devices. This has led to investigation of MD response in vicinity of nanostructures such as planar structures, nanospheres and metamaterials, using Green function approach^33^. Although enhancement of an MD source at the vicinity of subwavelength fiber has been observed experimentally^38^, there is no analytical model to investigate the interaction of MD in the vicinity of a nanofiber.

Furthermore, the interaction of a point source and a nanofiber is conceptually significant in the field of nanophotonics since localized strong light-matter interaction in one subwavelength dimension can be interfaced with the guided modes of nanowaveguides providing read-in/read-out access to the localized interaction. This creates the urge to investigate and develop an analytical model to study the MD and nanofiber interaction, within the frame work of magnetic dipole approximation in nanofiber based devices.

Here, we develop a model to study the interaction of a MD and an optical nanofiber. While models of ED-fiber systems based on both Green function^39,40^ and field decomposition methods^41–44^ have been reported, here, we develop our model based on the field decomposition method since it provides a better physical insight into the coupling of a MD emission into guided and radiation modes of the system. In particular, this method allows the decomposition of the guided and radiation modes into different polarisations and propagation directions of guided modes (hybrid, TE and TM modes) and radiation modes (TE- and TM-like) of a fiber, which cannot be achieved directly by employing Green function approach^39,40^. This method also enables us to not only calculate the ratio of spontaneous emission of a MD transition/source into fiber modes and free space (Purcell enhancement) but also identify parameters that allow higher MD emission coupling into the guided modes. This is vital in designing read-in/read-out channels in nanophotonic devices for monitoring localized interactions. As an example, monitoring the magnetic transition in NV (Nitrogen-vacancy) centers in nano-diamonds–doped within an optical fiber–has been used for remote localized magnetometry^45^.

## Results

### Mode decomposition method

The guided and radiation modes of a lossless ideal fiber with uniform cross section invariant along its length and uniform refractive index (no material absorption and dispersion) form a complete orthonormal base. For a fiber with radius *r*_co_ (*D* = 2*r*_co_) and refractive index *n*_co_ surrounded by an infinite region of refractive index *n*_cl_, these modes satisfy the following orthogonality relations:

1a $${\int }_{{A}_{\infty }}\,({{\bf{e}}}_{j}\times {{\bf{h}}}_{\nu }^{\ast })\cdot \hat{z}\,{\rm{d}}A={\int }_{{A}_{\infty }}\,({{\bf{e}}}_{j}^{\ast }\times {{\bf{h}}}_{\nu })\cdot \hat{z}\,{\rm{d}}A=2{N}_{j\nu }{\delta }_{j\nu },$$

1b $${N}_{j\nu }=\frac{1}{2}|{\int }_{{A}_{\infty }}\,({{\bf{e}}}_{j}\times {{\bf{h}}}_{\nu }^{\ast })\cdot \hat{z}\,{\rm{d}}A|;$$

1c $${\int }_{{A}_{\infty }}\,[{{\bf{e}}}_{j}(Q)\times {{\bf{h}}}_{\nu }^{\ast }(Q^{\prime} )]\cdot \hat{z}\,{\rm{d}}A={\int }_{{A}_{\infty }}\,[{{\bf{e}}}_{j}^{\ast }(Q^{\prime} )\times {{\bf{h}}}_{\nu }(Q)]\cdot \hat{z}\,{\rm{d}}A=2{N}_{j\nu }(Q){\delta }_{j\nu }\delta (Q-Q^{\prime} ),$$

1d $${N}_{j\nu }(Q)=\frac{1}{2}|{\int }_{{A}_{\infty }}\,[{{\bf{e}}}_{j}(Q)\times {{\bf{h}}}_{\nu }^{\ast }(Q^{\prime} )]\cdot \hat{z}\,{\rm{d}}A|.$$

Here, **e**_*j*_(*x*, *y*), **e**_*ν*_(*x*, *y*, *Q*), **h**_*j*_(*x*, *y*), **h**_*ν*_(*x*, *y*, *Q*) are electric guided, electric radiation, magnetic guided, and magnetic radiation modal vector fields, respectively and *N*_*jν*_ and *N*_*jν*_(*Q*) are guided and radiation normalization factors. The electric and magnetic fields of any system involving an optical fiber can be expanded based on the orthonormal base of the ideal fiber as:

2a $${\bf{E}}(x,y,z)=\sum _{j}\,{a}_{j}{{\bf{e}}}_{j}(x,y){e}^{i{\beta }_{j}z}+\sum _{\nu }\,{\int }_{0}^{\infty }\,{a}_{\nu }(Q){{\bf{e}}}_{\nu }(x,y,Q){e}^{i{\beta }_{\nu }(Q)z}dQ+BK,$$

2b $${\bf{H}}(x,y,z)=\sum _{j}\,{a}_{j}{{\bf{h}}}_{j}(x,y){e}^{i{\beta }_{j}z}+\sum _{\nu }\,{\int }_{0}^{\infty }{a}_{\nu }(Q){{\bf{h}}}_{\nu }(x,y,Q){e}^{i{\beta }_{\nu }(Q)z}dQ+BK,$$

in which *a*_*j*_, *a*_*ν*_(*Q*) are coupling coefficients for forward guided and radiation modes, respectively, *β*_*j*_ and *β*_*ν*_(*Q*) are respectively the corresponding guided mode and continuum/radiation propagation constants, modal parameter $Q=(D\mathrm{/2)(}{k}^{2}{n}_{cl}^{2}-{\beta }_{\nu }^{2}{)}^{\mathrm{1/2}}$, *D* is the core diameter, *k* = 2*π*/*λ*, and *BK* represents the contribution of backward guided and radiation modes. Guided modes have discrete propagation constants (*kn*_cl_ < *β*_*j*_ ≤ *kn*_co_) and are solutions to the wave equation in the step-index fiber. On the other hand propagating radiation modes span a continuum of propagation constants (0 ≤ *β*_*ν*_(*Q*) < *kn*_cl_).

The radiation fields in Eq. (2) include two contributions; propagating radiation fields, for which 0 < *Q* ≤ *Q*_max_ = *kn*_cl_*D*/2 (or 0 ≤ *β*_*ν*_(*Q*) < *kn*_cl_) and evanescent fields, for which *Q*_max_ < *Q* < ∞ (or *Re*(*β*) = 0; *Im*(*β*) > 0)^46^, [Chapter 25]. In our model, we only consider the propagating modes as they transmit the power along *z*. By finding the modal coupling coefficients, *a*_*j*_, *a*_*ν*_(*Q*) in Eq. (2), we can find the power coupled into each mode. The **e**_*j*_(*x*, *y*), **e**_*ν*_(*x*, *y*, *Q*), **h**_*j*_(*x*, *y*), **h**_*ν*_(*x*, *y*, *Q*) and *β*_*j*_ can be obtained by solving the wave equation in the core and cladding region and satisfying boundary conditions^43,46^. For the guided modes, this gives the expected step-index modal field expressions, which can be find in relevant textbooks^46^, and for radiation modes see the Supplementary Material.

While Eq. (2) is valid for any system, here we consider a system that contains a MD dipole in the vicinity of an optical fiber. A system of an electric dipole-fiber has been studied in details^43,44^ and coupling coefficients of guided and radiation modes have been derived (also reported here for completeness). The theory for a magnetic dipole-fiber system is given in details in the next part and Supplementary Materials.

### Electric dipole-fiber system

It has been demonstrated that for a system consist of an electric dipole (oscillating at frequency of *ω*) and a fiber, the coupling coefficient into the guided and radiation modes of the system can be obtained using following equations^44^:

3a $$|{a}_{j}{|}^{2}=\frac{{\omega }^{2}}{16{N}_{j}^{2}}|{{\bf{e}}}_{j}^{\ast }({{\bf{r}}}_{0})\cdot {\bf{p}}({{\bf{r}}}_{0}{)|}^{2};$$

3b $$|{a}_{\nu }(Q{)|}^{2}=\frac{{\omega }^{2}}{16{N}_{\nu }^{2}(Q)}|{{\bf{e}}}_{\nu }^{\ast }({{\bf{r}}}_{0},Q)\cdot {\bf{p}}({{\bf{r}}}_{0}{)|}^{2},$$

where **r**_0_ is the position of the electric dipole and **p** is the ED moment.

### Magnetic dipole-fiber system

A collection of magnetic dipoles lead to a magnetization **M**, which is defined as magnetic dipole moment per unit volume. In the case of one magnetic dipole (with a dipole moment **m**) oscillating at frequency *ω* and located at **r**_0_ = [*r*_0_, *θ*_0_, *z*_0_], the magnetization simplifies to:

4 $${\bf{M}}({\bf{r}})={\bf{m}}{e}^{-i\omega t}\delta ({\bf{r}}-{{\bf{r}}}_{0}\mathrm{)}.$$

**Figure 1:**
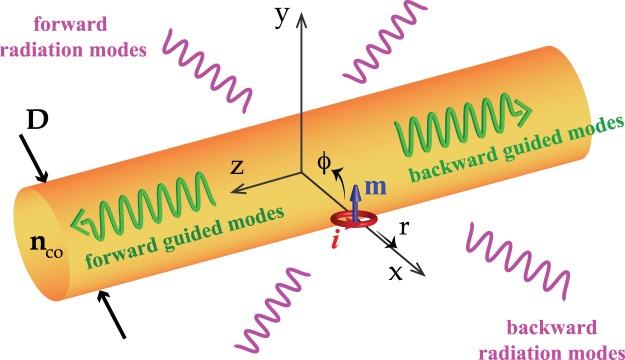
The schematic diagram of the hybrid system consist of a MD source and a dielectric fiber.

Here we do not consider the line-width of the dipole emission and assume that the emission is at a single frequency *ω*. Assuming that all fields are oscillating in time as *e*^−*iωt*^, Maxwell’s equations for the given system of MD dipole and nanofiber(Fig. 1) are^47^:

5a $$\nabla \times {\bf{E}}=-{\mu }_{0}\frac{\partial }{\partial t}{\bf{H}}-{\mu }_{0}\frac{\partial }{\partial t}{\bf{M}}{\boldsymbol{,}}$$

5b $$\nabla \times {\bf{H}}={\bf{J}}+\frac{\partial }{\partial t}{\bf{D}}{\boldsymbol{.}}$$

In order to find the coupling coefficients of dipole-fiber system, *a*_*j*_ and *a*_*ν*_(*Q*) in Eq. (2), we use the conjugated form of reciprocity theorem^46^ [Chapter 31]:

6 $$\frac{\partial }{\partial z}{\int }_{{A}_{\infty }}\,{\bf{F}}\cdot \hat{z}\,{\rm{d}}A={\int }_{{A}_{\infty }}\,\nabla \cdot {\bf{F}}\,{\rm{d}}A,$$

where ${\bf{F}}={\bf{E}}\times {\bar{{\bf{H}}}}^{\ast }+{\bar{{\bf{E}}}}^{\ast }\times {\bf{H}}$. In this equation **E** and **H** are the total fields of the dipole-fiber system, as defined in Eq. (2), with an associated magnetization **M** as defined in Eq. (4). The barred fields ($\bar{{\bf{E}}}$, $\bar{{\bf{H}}}$) correspond to fields of a particular but arbitrary guided or radiation mode *j* of a non absorbing fiber with no excitation (no MD source), i.e. $\bar{{\bf{E}}}={{\bf{e}}}_{j}{e}^{i{\beta }_{j}z},\,\bar{{\bf{H}}}={{\bf{h}}}_{j}{e}^{i{\beta }_{j}z}\,{\rm{or}}\,\bar{{\bf{E}}}={{\bf{e}}}_{\nu }(Q){e}^{i{\beta }_{\nu }z},\,\bar{{\bf{H}}}={{\bf{h}}}_{\nu }(Q){e}^{i{\beta }_{\nu }z}$. The conjugated form of reciprocity theorem has been used since we only consider lossless waveguides. For highly lossy waveguides such as metallic or hybrid metallic-dielectric waveguides one needs to consider the unconjugated form of reciprocity theorem^46^ [Chapter 31].

Substituting the electric and magnetic fields of perturb (MD-fiber) and non-perturb (fiber only) systems in Eq. (6) and implementing the orthogonality of the modes, Eq. (6) simplifies to:

7 $${a}_{j}(z)=\frac{1}{4{N}_{j}}{e}^{i\omega t}{\int }_{V}\,{{\bf{h}}}_{j}^{\ast }.(-\,{\mu }_{0}\frac{\partial }{\partial t}{\bf{M}})\exp (-\,i{\beta }_{j}z)dV.$$

Considering single MD excitation Eq. (4), we find the coupling power to guided and radiation modes respectively, as:

8a $${P}_{j}=|{a}_{j}{|}^{2}\,{N}_{j},$$

8b $${P}_{\nu }(Q)=|{a}_{\mu }(Q{)|}^{2}{N}_{\nu }(Q)$$

where |*a*_*j*_|^2^ and |*a*_*μ*_(*Q*)|^2^ are given by:

9a $$|{a}_{j}{|}^{2}=\frac{{\omega }^{2}{\mu }_{0}^{2}}{16{N}_{j}^{2}}|{{\bf{h}}}_{j}^{\ast }({{\bf{r}}}_{0})\cdot {\bf{m}}({{\bf{r}}}_{0}{)|}^{2},$$

9b $$|{a}_{\nu }(Q{)|}^{2}=\frac{{\omega }^{2}{\mu }_{0}^{2}}{16{N}_{\nu }^{2}(Q)}|{{\bf{h}}}_{\nu }^{\ast }({{\bf{r}}}_{0},Q)\cdot {\bf{m}}({{\bf{r}}}_{0}{)|}^{2}.$$

Consequently the total power of the system is given by:

10 $${P}_{{\rm{total}}}=\sum _{j}\,{P}_{j}+\sum _{\nu }\,{\int }_{0}^{{Q}_{{\rm{\max }}}}\,{P}_{\nu }(Q)\,{\rm{d}}Q.$$

The field decomposition method also enables us to separate the radiation modes into TE and TM like radiation modes^46^, fields with strong *h*_z_ and *e*_z_ components respectively. This will enable us to understand the nature of the radiations peaks and consequently to explain the overlapping of the position of the peaks with whispering gallery modes (WGM) of a disk. Moreover, the field decomposition method allows us breaking down the integration over the continuum of propagation constant of radiation modes, Eq. (10), and investigating the distribution of the power over different intervals at the vicinity of the radiation peak.

**Figure 2:**
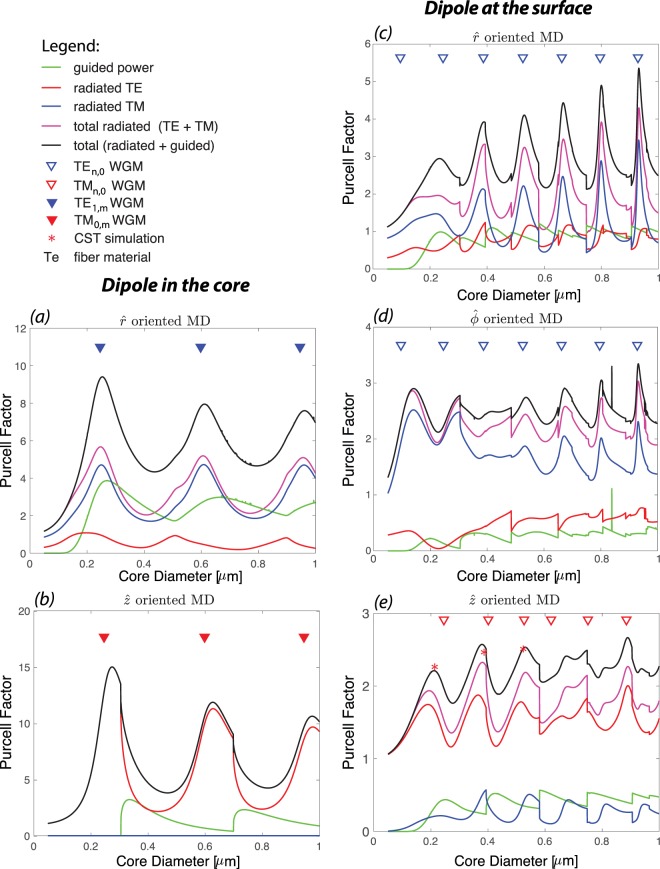
Purcell factor of a MD and Te fiber system (*n*_co_ = 2.025): TE (red), TM (blue) and total (magenta) radiated power, guided power (green) and total power (in black). Two configurations are considered: MD at the center of the fiber, (**a,b**), and MD at the core-cladding interface, (**c–e**). For each configuration three different orientations ($\hat{r}$, $\hat{\phi }$, and $\hat{z}$) are analyzed. Note that for the MD in the core configuration the plot for $\hat{\varphi }$ is omitted as there is no distinction between $\hat{r}$ and $\hat{\phi }$ orientations. All the powers are normalized to the total power emitted by a MD in the air. The triangles demonstrate the WGM peak positions (TE WGM in blue and TM WGM is red) of a 2D Te disk and the red asterisks, which represents the normalised total power of the system, are calculated using numerical simulation.

Here, we consider a linearly polarized MD excited at *λ* = 700 nm and located at the vicinity of a tellurite (Te) fibre, to investigate the guided, radiated and total Purcell factor (normalised power) of the hybrid system, Fig. 2. Note that all the powers are normalized to the total power emitted by a MD in the air (*P*_0_ = *μ*_0_*m*^2^*ω*^4^/12*πc*^3^ for a MD with a moment *m*)^47^. As expected, we observe that the relative dipole position with respect to fiber and dipole orientation affect the Purcell factor of guided and radiated modes. In general, we observe that when MD is at core-cladding interface the total radiated power dominates the guided power, however when MD is in the center of the fiber there are diameters ($\hat{r}-$oriented MD) where Purcell factor of guided modes can be larger than that of radiated modes. This information will be critical in defining the desired fiber parameters in nanophotonics devices depending whether strong coupling into the guided modes or radiation modes is required.

**Figure 3:**
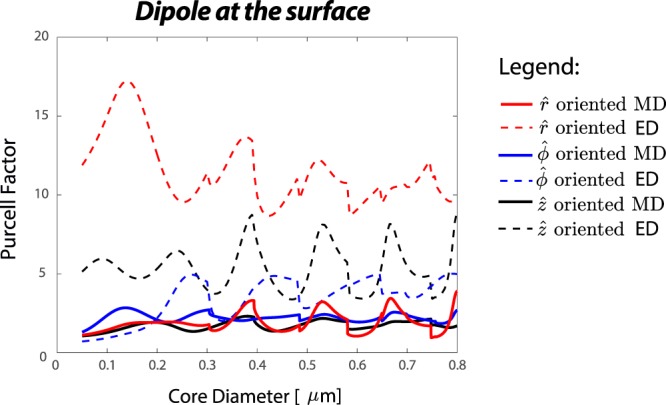
Purcell factor of total radiated power from a hybrid system consist of a MD/ED and a Te fiber (*n*_co_ = 2.025): when dipole source is oriented along $\hat{r}$ (red), $\hat{\varphi }$ (blue), and $\hat{z}$ (black).

We observe that shape and position of the peaks of the total radiated power is defined by either TE or TM radiated modes similar to ED excitation^25^. This can be explained in terms of the excitation fields. For example, a $\hat{z}$−oriented MD on the surface of fiber (see Fig. 2(c)) couples strongly into a mode with a large *z*−component magnetic field (maximum projection see Eq. (9b)); thus it mainly couples into TE radiated modes of the fiber (*H*_r_, *E*_ϕ_, *H*_z_). We also witness that the positions of these radiation peaks overlap with the position of the fundamental TM whispering gallery mode (TM-WGM) resonances (red triangles in Fig. 2e) of a two-dimensional (2D) microdisk calculated independently by solving Maxwell’s equations for a diameter equal to that of the fiber^25,44^. The TM-WGMs are modes that propagate in $\hat{\varphi }$ direction and have no magnetic field component in that direction (non-zero fields are: *H*_r_, *E*_ϕ_, *H*_z_). The CST numerical simulation also validates almost similar enhancement (the red asterisks in Fig. 2(e)) observed by analytical model for the total power and the formation of WGMs in the fiber cross-section (discussed later and shown in inset of Fig. 5). Similarly, $\hat{r}$−or $\hat{\varphi }$−oriented MD on the surface of the fiber (see Fig. 2(c,d)) mainly couples into TM radiated modes of the fiber (mode with only transverse magnetic component), and the positions of the peaks overlap with the positions of the fundamental TE whispering gallery mode (TE-WGM) resonances (blue triangles in Fig. 2(c,d)) of a microdisk. We notice that the Purcell enhancement of total/radiation power increases and the peaks get narrower for larger fiber diameters. This trend is consistent with the WGM resonances of a microdisk, i.e. for larger diameter the resonances get stronger and narrower^44^. In general, the Purcell enhancement of an ED near the fiber is stronger than that of a MD^25^. Comparing the radiated Purcell enhancement (Fig. 3) at the position of the first peak for similar orientations reveals that the first peak of a *r*−, *ϕ*− or *z*−oriented ED is more than 9, 1.5 or 3 times stronger than that of same oriented MD near the fiber. However, the enhancement for a *ϕ*−oriented MD near the fiber dominates that of a *ϕ*−oriented ED when the diameter of the fiber is smaller than 200 *nm* (*D*/*λ* < 0.28). This is due to preferential coupling of a *ϕ*−oriented MD to TM like radiated modes.

The MD-fiber system behaves differently, when the dipole is in the center of the fiber. There are fiber diameters, at which the Purcell factor of guided modes are equal or larger than that of radiated modes (Fig. 2(a,b)). Note that because of symmetry there is no distinction between $\hat{r}$ and $\hat{\varphi }$ orientations when the MD is in the center of the fiber. Hence we opt to only display the $\hat{r}$ direction outputs. Similar to when a MD is positioned on the fiber surface, the shape and position of the peaks of the total radiated power are defined by either TE or TM radiated modes, which can be explained in terms of the excitation fields. Note that a $\hat{z}$−oriented MD (Fig. 2(b)) only couples into the TE radiated modes since this is the only mode that has non-zero *z*−component magnetic field. The distinctive features of the peaks for MD in the center of the fiber are the descent in Purcell factor with the diameter increase, the wider separation between them and the excitation of higher order radial modes of TM_0,m_ and TE_1,m_ WGMs. When the source is at the center of the fiber, **r**_0_ = 0 in Eq. (4), the argument of Bessel functions for the electromagnetic fields of the system [see supplementary] are zero. The only non-zero Bessel functions at the origin are those with the azimuthal mode number *ν* = 0, i.e., *J*_0_(0) or ${J}_{1}^{\text{'}}\mathrm{(0)}$. As a result, higher order radial modes that are usually much smaller in comparison with higher order azimuthal modes appear in the spectrum. This explains why the position of the peaks (Fig. 2(a,b)) in these cases overlap with higher order radial modes of TE_1,m_ and TM_0,m_ WGMs. It also explains the wider separation of the peaks and their reduced Purcell factor–as core diameter increases–since the overlap of the fields of the MD and higher order radial modes decreases.

**Figure 4:**
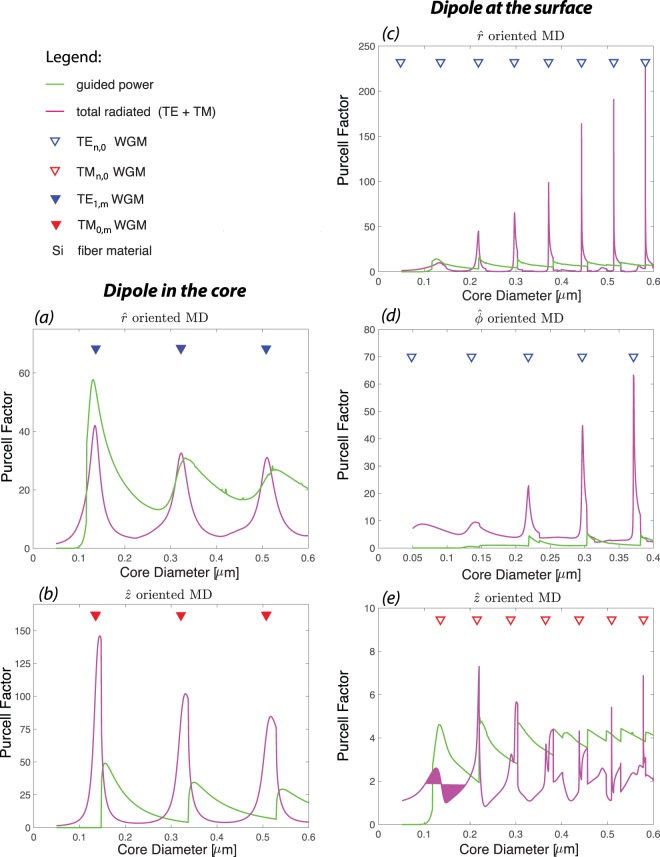
Purcell factor of a hybrid system consist of a MD and a Si fiber (*n*_co_ = 3.778): the total radiated (magenta) and guided (green) powers. Two configurations are considered: MD at the center of the fiber, (**a,b**), and MD at the core-cladding interface, (**c–e**). For each configuration three different orientations ($\hat{r}$, $\hat{\varphi }$, and $\hat{z}$) are analyzed. Note that for the MD in the core configuration the plot for $\hat{\varphi }$ is omitted as there is no distinction between $\hat{r}$ and $\hat{\varphi }$ orientations. All the powers are normalized to the total power emitted by a MD in the air. The triangles demonstrate the WGM peak positions (TE WGM in blue and TM WGM is red) of a 2D Si disk.

We observe that increasing the refractive index of the fiber (in this case to silicon, Si) increases the Purcell factor of the magnetic dipole (see Fig. 4), which is congruent with enhancement of ED and MD Purcell factors at the vicinity of high permittivity nanoparticles^33,43^. There are a few factors contributing into this enhancement as follows. Increasing the refractive index of the fiber leads into tighter confinement of the guided modes and enhancement of field intensity at the core-cladding interface^48^. Higher refractive index also leads into small mode volumes and higher quality factor whispering gallery modes^49^. As a consequence, higher Purcell factor can be achieved. Note that changing the host material of the fiber to higher permittivity results into the blue shift and densening of resonances (Fig. 4). We also observe that the Purcell factor of guided mode can be greater than that of the radiated modes when the host material of the fiber has high permittivity. This is specifically more prominence for a *z*−oriented MD at the core-cladding interface (Fig. 4(a)) and a *r*−oriented MD in the center of the fiber (Fig. 4(e)). The radiation peaks have dominant TE or TM radiated modes contribution similar to Te fiber case, which can be explained in terms of the excitation fields.

A careful examination of the total radiated power reveals that the peaks have asymmetric profiles. The first a few peaks have steeper trailing edge compared to their leading edge, while, this becomes opposite (steeper leading edge compared to their trailing edges) for the rest of the peaks. This can be explained by breaking down the radiated power into small intervals of *β*(*Q*)/*k*. As discussed earlier, the total radiated power is calculated by integrating over the propagating radiated power density from 0 to *Q*_max_, Eq. (10), which for air cladding fiber is associated with $0\le \beta (Q)\le {\beta }_{{\rm{\max }}}=k$. The *β*(*Q*) is the propagation constant of radiated modes along the *z*-direction. The radiation power with values of *β*(*Q*) → 0 are completely contained within the transverse plane, while with values of $0 < \beta (Q)\le k$ consist of non-transverse radiation mode, known as skew modes.

**Figure 5:**
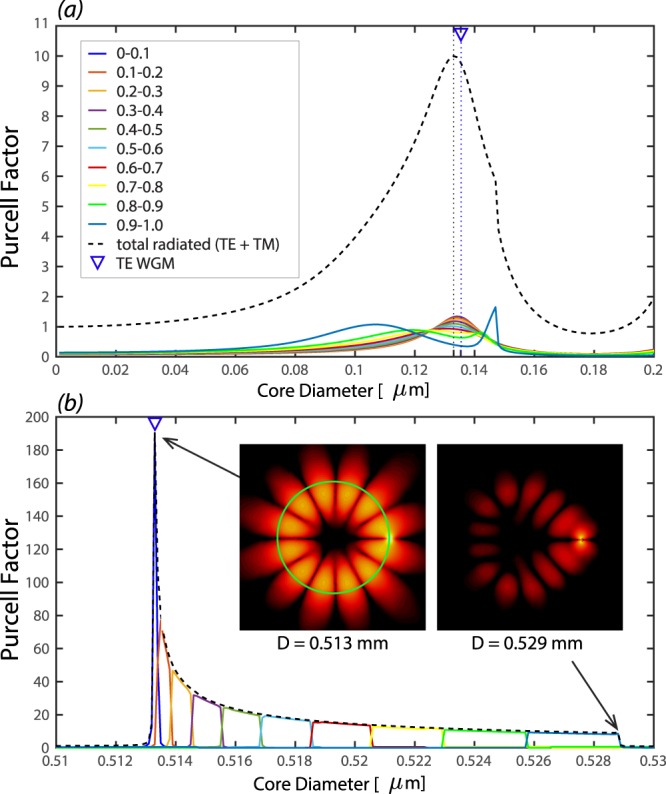
The breakdown of the Purcell factor of the radiated power of a *r*−oriented MD located at the interface of a Si-fiber in terms of 0.1 intervals of *β*(*Q*)/*k*: at the position of (**a**) the first peak and (**b**) the sixth peak in Fig. 4(c). Total radiated power (black dashed line) is consist of the summation of all these curves. The insets in (**b**) show the average electric field (*z*−component) at the fiber cross section in *z* = 0 plane, indicating the formation of a WGM (TE_6,0_) with 2 × 6 = 12 lobes. The green circle represents the core-cladding interface and the MD is the bright spot on the right hand side.

To explain the different asymmetries observed in the radiation peaks, we break down the total radiated power into small intervals of *β*(*Q*)/*k* = 0.1 (here after called as *β* bins) for the first and sixth peaks (in Fig. 4(c)) of a *r*−oriented MD located at the interface of a Si-fiber as shown in Fig. 5(a,b), respectively. Overall, as expected, the total radiation peaks are the envelopes of underlying radiation peaks with different *β* bins, i.e. radiation modes propagating with different *z*−component wavevector. Moreover, the position of the peaks (vertical dotted black line that overlaps with TE-WGMs for higher order peaks and is slightly shifted for the first peaks) are mainly due to the contribution of radiation power associated with the close to zero bins of *β*. We observe that lower *β* bins have higher peaks for higher order radiation peaks (Fig. 5(b)), while the height of the *β* bins peaks are at a similar range for the first radiation peak (Fig. 5(a)). The main distinction between two peaks in Fig. 5 becomes apparent for higher *β* bin values, i.e. the shift of the curves to opposite directions, which explains the difference between leading and trailing edges of radiation peaks. The curves associated with higher *β* bin values are broader and are responsible for the asymmetry profile of the overall peak. This is consistent with the asymmetry observed due to existence of the skew rays using Green tensor formalism in a circular dielectric waveguide^40^.

As indicated earlier, the radiations peaks become narrower and higher as the diameter of the fiber increases for a MD on the surface of the fiber. We observe that for larger diameters, a smaller range of the *β* bin contributes into radiation peaks. This indicates that these peaks have a very small contribution from non-transverse propagating modes (skew modes) and is almost purely radiating in the transverse plane. At the resonance, the radiation is localized within a transverse plane normal to the fiber axis and at the position of the dipole and we observe the formation of WGM in the core cross section, as shown in the inset of Fig. 5(b), while this is not the case away from the resonance. This is similar to ED-fiber system where a self-formed cavity appears for higher order radiation modes^44^ or the case of a dielectric planar microcavity in which a localized mode forms in spite of no lateral confinement^50^.

In summary, we have presented a full-vectorial model of the radiation of a magnetic dipole (MD) in the vicinity of a nanofiber. Using a field decomposition method, we have provided insight into the coupling of a linearly polarized MD emission into guided and radiated modes of a step-index optical fiber. The field decomposition method has enabled us to identify diameters/wavelengths, where the emission power–either collected by guided modes or radiated by the fiber–can be larger than the total power emitted by a MD source. This is a key information in designing fiber based nanophotonic devices, e.g. nanoantenna, nanolasers, and read-in/read-out access waveguides for quantum signal processing. This versatile approach has also allowed us to determine the power distribution among different traveling waves that constitute the overall radiated power and hence explain the asymmetric profile of the radiated peaks. Note that the radiated peaks have been obtained for lossless fibers and hence their *Q*-factors are purely due to radiation process. The effect of fiber material loss (*α*_m_) on degradation of the *Q*-factors of the peaks can be included by modifying 1/*Q* with 1/*Q* + 1/*Q*_m_, where *Q*_m_ = 2*πn*/*λα*_m_ is the *Q*-factor due to material loss^51^. As a future work, using our approach, one is able to investigate the potential asymmetry in the power distribution of a MD-fiber system into forward and backward guided and radiated modes due to chiral properties of modes of a nanofiber.

## Methods

Numerical analysis is performed using CST Microwave Studio. The CST numerical simulation has been used to validate the in-house developed codes of our proposed analytical-numerical approach. Two different solver modules are used, time domain solver based on finite integration technique and frequency domain solver based on finite element method. Two different MD sources operating at 700 nm are considered, CST macro magnetic dipole source and a sub-wavelength loop excited by a current source. The former is a small flat patch that only works with time domain solver and the later is an eight nanometer loop excited by a current source that works with both solvers. Both sources can be used when dipole is either inside or outside the fiber. However, the source should be chosen carefully, when the MD source is at the core-cladding interface. Although dipole sources are considered point sources, they have physical area. In order to get consistent results, the physical area of the source should completely be in the cladding medium for the case of dipole on the interface. This is achieved by using the CST macro (flat patch) when the MD source is aligned in either *ϕ*−or *z*−directions, and the sub-wavelength loop when the MD source is aligned in *r*−direction.

The system under study (MD source and fiber) is constructed so that the fiber was along *z*−direction and the center of the fiber is at the center of coordinates (see Fig. 1). The MD source is replaced on the fiber interface on *x*−axis. CST open boundary conditions (a perfectly matched layer with minimum reflection) is used in the *z*−direction to represent infinitely long fiber. Two spherical shells with radius of half and quarter of fiber length, which are concentric with fiber, are considered for calculating the total power emitted from the system. Two simulations are conducted, MD-fiber coupled system and MD only (where the fiber is replaced by air). The simulations are repeated for four fiber lengths (3, 4, 5 and 6 *μ*m) and we observe that the calculated Purcell factors are almost similar. The data represented is the averaged total power integrated on the spherical shells and different fiber lengths.

## Supplementary information

Supplementary material

## References

[1] Afshar VS, Warren-Smith SC, Monro TM (2007). Enhancement of fluorescence-based sensing using microstructured optical fibres. Opt. Express.

[2] Atakaramians S, Afshar VS, Fischer BM, Abbott D, Monro TM (2008). Porous fibers: a novel approach to low loss thz waveguides. Opt. Express.

[3] Afshar VS, Monro TM (2009). A full vectorial model for pulse propagation in emerging waveguides with subwavelength structures part i: Kerr nonlinearity. Opt. Express.

[4] Tong L, Zi F, Guo X, Lou J (2012). Optical microfibers and nanofibers: A tutorial. Opt. Commun..

[5] Shin H (2013). Tailorable stimulated brillouin scattering in nanoscale silicon waveguides. Nat. Commun..

[6] Xu F, Xing Wu Z, Qing Lu Y (2017). Nonlinear optics in optical-fiber nanowires and their applications. Prog. Quantum Electron..

[7] Nielsen, M. P., Shi, X., Dichtl, P., Maier, S. A. & Oulton, R. F. Giant nonlinear response at a plasmonic nanofocus drives efficient four-wave mixing. *Science* **358**, 1179–1181, 10.1126/science.aao1467, http://science.sciencemag.org/content/358/6367/1179.full.pdf (2017). 10.1126/science.aao1467 29191907

[8] Afshar VS, Zhang WQ, Ebendorff-Heidepriem H, Monro TM (2009). Small core optical waveguides are more nonlinear than expected: experimental confirmation. Opt. Lett..

[9] Kivshar Y, Miroshnichenko A (2017). Meta-optics with Mie resonances. Opt. Photonics News.

[10] Lodahl P (2017). Chiral quantum optics. Nature.

[11] Schuller JA, Zia R, Taubner T, Brongersma ML (2007). Metamaterials based on electric and magnetic resonances of silicon carbide particles. Phys. Rev. Lett..

[12] Peng L (2007). Experimental Observation of Left-Handed Behavior in an Array of Standard Dielectric Resonators. Phys. Rev. Lett..

[13] Schuller JA, Zia R, Taubner T, Brongersma ML (2007). Dielectric Metamaterials Based on Electric and Magnetic Resonances of Silicon Carbide Particles. Phys. Rev. Lett..

[14] Zhao Q (2008). Tunable negative permeability in an isotropic dielectric composite. Appl. Phys. Lett..

[15] Popa BI, Cummer SA (2008). Compact dielectric particles as a building block for low-loss magnetic metamaterials. Phys. Rev. Lett..

[16] Vynck K (2009). All-dielectric rod-type metamaterials at optical frequencies. Phys. Rev. Lett..

[17] Zhao Q, Zhou J, Zhang F, Lippens D (2009). Mie resonance-based dielectric metamaterials. Mater. Today.

[18] Evlyukhin AB, Reinhardt C, Seidel A, Lukýanchuk BS, Chichkov BN (2010). Optical response features of Si-nanoparticle arrays. Phys. Rev. B.

[19] Ginn JC (2012). Realizing optical magnetism from dielectric metamaterials. Phys. Rev. Lett..

[20] Kuznetsov AI, Miroshnichenko AE, Fu YH, Zhang J, Lukyanchuk B (2012). Magnetic light. Sci. Reports.

[21] Fu YH, Kuznetsov AI, Miroshnichenko AE, Yu YF, Lukyanchuk B (2013). Directional visible light scattering by silicon nanoparticles. Nat. Commun..

[22] Staude I (2013). Tailoring directional scattering through magnetic and electric resonances in subwavelength silicon nanodisks. ACS Nano.

[23] Bi K (2014). Magnetically tunable Mie resonance-based dielectric metamaterials. Sci. Reports.

[24] Habteyes T (2014). Near-field mapping of optical modes on all-dielectric silicon nanodisks. ACS Photonics.

[25] Atakaramians S (2016). Strong magnetic response of optical nanofibers. ACS Photonics.

[26] Le Feber, B., Rotenberg, N. & Kuipers, L. Nanophotonic control of circular dipole emission. *Nat. Commun*. **6**, 10.1038/ncomms7695 Cited By 49 (2015). 10.1038/ncomms7695 25833305

[27] Mitsch R, Sayrin C, Albrecht B, Schneeweiss P, Rauschenbeutel A (2014). Quantum state-controlled directional spontaneous emission of photons into a nanophotonic waveguide. Nat. Commun..

[28] Sayrin, C. *et al*. Nanophotonic optical isolator controlled by the internal state of cold atoms. *Phys. Rev. X* **5**, 10.1103/PhysRevX.5.041036 Cited By 38 (2015).

[29] Petersen, J., Volz, J. & Rauschenbeutel, A. Chiral nanophotonic waveguide interface based on spin-orbit interaction of light. *Science* **346**, 67–71, 10.1126/science.1257671 Cited By 139 (2014). 10.1126/science.1257671 25190718

[30] Lukas, N. & Bert, H. *Principles of Nano-Optics* (Cambridge University Press, 2006).

[31] Kasperczyk M, Person S, Ananias D, Carlos LD, Novotny L (2015). Excitation of magnetic dipole transitions at optical frequencies. Phys. Rev. Lett..

[32] Smirnova D, Kivshar YS (2016). Multipolar nonlinear nanophotonics. Optica.

[33] Baranov, D., Savelev, R., Li, S., Krasnok, A. & Alu, A. Modifying magnetic dipole spontaneous emission with nanophotonic structures. *Laser Photonics Rev*. **11**, 10.1002/lpor.201600268 (2017).

[34] Thommen Q, Mandel P (2006). Left-handed properties of erbium-doped crystals. Opt. letters.

[35] Liu Y, Tu D, Zhu H, Chen X (2013). Lanthanide-doped luminescent nanoprobes: controlled synthesis, optical spectroscopy, and bioapplications. Chem. Soc. Rev..

[36] Zurita-Sánchez JR, Novotny L (2002). Multipolar interband absorption in a semiconductor quantum dot. II. Magnetic dipole enhancement. JOSA B.

[37] Fang, X., Tseng, M. L., Tsai, D. P. & Zheludev, N. I. Coherent excitation-selective spectroscopy of multipole resonances. *Phys. Rev. Appl*. **5**, 10.1103/PhysRevApplied.5.014010 (2016).

[38] Atakaramians S (2018). Enhanced terahertz magnetic dipole response by subwavelength fiber. APL Photonics.

[39] Søndergaard T, Tromborg B (2001). General theory for spontaneous emission in active dielectric microstructures: Example of a fiber amplifier. Phys. Rev. A.

[40] Fussell, D., McPhedran, R. & Martijn de Sterke, C. Decay rate and level shift in a circular dielectric waveguide. *Phys. Rev. A* **71**, 10.1103/PhysRevA.71.013815 (2005). 10.1103/PhysRevE.72.046605 16383552

[41] Żakowicz W, Janowicz M (2000). Spontaneous emission in the presence of a dielectric cylinder. Phys. Rev. A.

[42] Klimov VV, Ducloy M (2004). Spontaneous emission rate of an excited atom placed near a nanofiber. Phys. Rev. A.

[43] Henderson MR, Afshar VS, Greentree AD, Monro TM (2011). Dipole emitters in fiber: interface effects, collection efficiency and optimization. Opt. Express.

[44] Afshar, V. S., Henderson, M. R., Greentree, A. D., Gibson, B. C. & Monro, T. M. Self-formed cavity quantum electrodynamics in coupled dipole cylindrical-waveguide systems. *Opt. Express* **22**, 11301, 10.1364/OE.22.011301 (2014). 10.1364/OE.22.011301 24921827

[45] Ruan Y (2018). Magnetically sensitive nanodiamond-doped tellurite glass fibers. Sci. Reports.

[46] Snyder, A. W. & Love, J. *Optical Waveguide Theory*, 1st edition edn. (Chapman and Hall Ltd, 1983).

[47] Griffiths, D. & College, R. *Introduction to Electrodynamics* (Pearson, 2008).

[48] Monro TM (2007). Beyond the diffraction limit. Nat. Photonics.

[49] Hall JM (2017). Unified theory of whispering gallery multilayer microspheres with single dipole or active layer sources. Opt. Express.

[50] Björk G, Machida S, Yamamoto Y, Igeta K (1991). Modification of spontaneous emission rate in planar dielectric microcavity structures. Phys. Rev. A.

[51] Reynolds T (2015). Unified theory of whispering gallery multilayer microspheres with single dipole or active layer sources. Opt. Express.

