# Supplementary material for: Radiated and guided optical waves of a magnetic dipole-nanofiber system
Source: Sci Rep. 2019 Mar 5;9:3568. doi: 10.1038/s41598-018-38115-z (PMC6401418; doi:10.1038/s41598-018-38115-z)
Supplement: Supplementary file 1 — Supplementary material [file 41598_2018_38115_MOESM1_ESM.pdf]

Supplementary Materials

Radiated and guided optical waves of a magnetic dipole-nanofiber system

Shaghik Atakaramians[1,2,*], **Feng Q. Dong**[2,**], **Tanya M. Monro**[3,+], **and Shahraam Afshar V.**[3,++]

[1]School of Electrical Engineering and Telecommunications, UNSW Sydney, Sydney, NSW 2052, Australia

[2]Institute of Photonics and Optical Science, School of Physics, The University of Sydney, Sydney, NSW 2006, Australia

[3]Laser Physics and Photonic Devices Laboratories, School of Engineering, University of South Australia, Mawson Lakes, SA 5095, Australia

[*]s.atakaramians@unsw.edu.au,T:+61431465884., F:+61293855993

[**]fengqiu.dong@gmail.com

[+]tanya.monro@unisa.edu.au

[++]shahraam.afshar@unisa.edu.au

ABSTRACT

Here we calculate the radiation modes of a step index fiber, where we derive all the electric and magnetic components.

Radiation modes of a step index fiber

To calculate the radiation modes, we separate the fields into two terms, one representing the field in the absence of the waveguide (‘free-space’) and a second term that includes scattering from the waveguide.[1] The z components of radiation modes are then of the following form considering the boundary conditions

and avoiding the singularities of Bessel functions:[1]

$$e_z = \begin{cases} a_\nu J_\nu(UR) f_\nu(\theta) & 0 \le r < r_{\text{co}} \\ \left[c_\nu^f J_\nu(QR) + c_\nu^s H_\nu^{(1)}(QR)\right] f_\nu(\theta) & r_{\text{co}} \le r < \infty \end{cases} \tag{1}$$

$$h_z = \begin{cases} b_\nu J_\nu(UR) g_\nu(\theta) & 0 \le r < r_{\text{co}} \\ \left[d_\nu^f J_\nu(QR) + d_\nu^s H_\nu^{(1)}(QR)\right] g_\nu(\theta) & r_{\text{co}} \le r < \infty \end{cases} \tag{2}$$

$$f_\nu(\theta) = \begin{cases} \cos(\nu\theta) & \text{even modes} \\ \sin(\nu\theta) & \text{odd modes} \end{cases} \tag{3}$$

$$g_\nu(\theta) = \begin{cases} -\sin(\nu\theta) & \text{even modes} \\ \cos(\nu\theta) & \text{odd modes} \end{cases} \tag{4}$$

where ν is an azimuthal mode index, $Q = (D/2)(k^2 n_{cl}^2 - \beta^2)^{1/2}$, the superscripts f and s denote the 'free-space' and scattering terms, respectively, and a_ν, b_ν, c_ν^f, c_ν^s, d_ν^f and d_ν^s are constants that can be determined by applying the continuity conditions at the core-cladding interface (see Table 1, which is taken from Ref. [1, page 525]). The four other components of the electric and magnetic field, (e_r, e_θ, h_r and h_θ), can be expressed in terms of the derivatives of e_z and h_z as:

$$\begin{pmatrix} e_r \\ h_\theta \end{pmatrix} = \frac{i}{k^2 n^2 - \beta^2} \begin{pmatrix} \beta & \left(\frac{\mu_0}{\varepsilon_0}\right)^{\frac{1}{2}} k \\ \left(\frac{\varepsilon_0}{\mu_0}\right)^{\frac{1}{2}} k n^2 & \beta \end{pmatrix} \begin{pmatrix} \frac{\partial e_z}{\partial r} \\ \frac{1}{r}\frac{\partial h_z}{\partial \theta} \end{pmatrix}$$

$$\begin{pmatrix} e_\theta \\ h_r \end{pmatrix} = \frac{i}{k^2 n^2 - \beta^2} \begin{pmatrix} \beta & -\left(\frac{\mu_0}{\varepsilon_0}\right)^{\frac{1}{2}} k \\ -\left(\frac{\varepsilon_0}{\mu_0}\right)^{\frac{1}{2}} k n^2 & \beta \end{pmatrix} \begin{pmatrix} \frac{1}{r}\frac{\partial e_z}{\partial \theta} \\ \frac{\partial h_z}{\partial r} \end{pmatrix}$$

Substituting the expressions for e_z and h_z, Eqs. (1) and (2), into the above equations, (5), gives:

For $0 \leq r < r_{\text{co}}$:

$$e_r = \frac{f_\nu(\theta)}{k^2 n_{\text{core}}^2 - \beta^2} \frac{iU}{2r_{\text{co}}} \left\{ \beta a_\nu \left[J_{\nu-1}(UR) - J_{\nu+1}(UR) \right] - \left(\frac{\mu_0}{\varepsilon_0} \right)^{\frac{1}{2}} k b_\nu \left[J_{\nu-1}(UR) + J_{\nu+1}(UR) \right] \right\} \quad (5)$$

$$e_\theta = \frac{g_\nu(\phi)}{k^2 n_{\text{core}}^2 - \beta^2} \frac{iU}{2r_{\text{co}}} \left\{ \beta a_\nu \left[J_{\nu-1}(UR) + J_{\nu+1}(UR) \right] - \left(\frac{\mu_0}{\varepsilon_0} \right)^{\frac{1}{2}} k b_\nu \left[J_{\nu-1}(UR) - J_{\nu+1}(UR) \right] \right\} \quad (6)$$

$$h_r = \frac{-g_\nu(\phi)}{k^2 n_{\text{core}}^2 - \beta^2} \frac{iU}{2r_{\text{co}}} \left\{ \left(\frac{\varepsilon_0}{\mu_0} \right)^{\frac{1}{2}} k^2 n_{\text{core}}^2 a_\nu \left[J_{\nu-1}(UR) + J_{\nu+1}(UR) \right] - \beta b_\nu \left[J_{\nu-1}(UR) - J_{\nu+1}(UR) \right] \right\} \quad (7)$$

$$h_\theta = \frac{f_\nu(\theta)}{k^2 n_{\text{core}}^2 - \beta^2} \frac{iU}{2r_{\text{co}}} \left\{ \left(\frac{\varepsilon_0}{\mu_0} \right)^{\frac{1}{2}} k n_{\text{core}}^2 a_\nu \left[J_{\nu-1}(UR) - J_{\nu+1}(UR) \right] - \beta b_\nu \left[J_{\nu-1}(UR) + J_{\nu+1}(UR) \right] \right\} \quad (8)$$

For $r_{\text{co}} \leq r < \infty$

$$e_r = \frac{f_\nu(\phi)}{k^2 n_{\text{clad}}^2 - \beta^2} \frac{iQ}{2r_{\text{co}}} \left(\beta \left\{ c_\nu^f \left[J_{\nu-1}(QR) - J_{\nu+1}(QR) \right] + c_\nu^s \left[H_{\nu-1}^{(1)}(QR) - H_{\nu+1}^{(1)}(QR) \right] \right\} - k \left(\frac{\mu_0}{\varepsilon_0} \right)^{\frac{1}{2}} \left\{ d_\nu^f \left[J_{\nu-1}(QR) - J_{\nu+1}(QR) \right] + d_\nu^s \left[H_{\nu-1}^{(1)}(QR) - H_{\nu+1}^{(1)}(QR) \right] \right\} \right) \quad (9)$$

$$
e_\theta = \frac{g_\nu(\phi)}{k^2 n_{\text{clad}}^2 - \beta^2} \frac{iQ}{2r_{\text{co}}} \left(\beta \left\{ c_\nu^f \left[J_{\nu-1}(QR) + J_{\nu+1}(QR) \right] + c_\nu^s \left[H_{\nu-1}^{(1)}(QR) + H_{\nu+1}^{(1)}(QR) \right] \right\} \right.
$$

$$
\left. -k \left(\frac{\mu_0}{\varepsilon_0} \right)^{\frac{1}{2}} \left\{ d_\nu^f \left[J_{\nu-1}(QR) + J_{\nu+1}(QR) \right] + d_\nu^s \left[H_{\nu-1}^{(1)}(QR) + H_{\nu+1}^{(1)}(QR) \right] \right\} \right) \quad (10)
$$

$$
h_\theta = \frac{f_\nu(\phi)}{k^2 n_{\text{clad}}^2 - \beta^2} \frac{iQ}{2r_{\text{co}}} \left(\left(\frac{\varepsilon_0}{\mu_0} \right)^{\frac{1}{2}} k n_{\text{clad}}^2 \left\{ c_\nu^f \left[J_{\nu-1}(QR) - J_{\nu+1}(QR) \right] + c_\nu^s \left[H_{\nu-1}^{(1)}(QR) - H_{\nu+1}^{(1)}(QR) \right] \right\} \right.
$$

$$
\left. -\beta \left\{ d_\nu^f \left[J_{\nu-1}(QR) - J_{\nu+1}(QR) \right] + d_\nu^s \left[H_{\nu-1}^{(1)}(QR) - H_{\nu+1}^{(1)}(QR) \right] \right\} \right) \quad (11)
$$

$$
h_r = \frac{-g_\nu(\phi)}{k^2 n_{\text{clad}}^2 - \beta^2} \frac{iQ}{2r_{\text{co}}} \left(\left(\frac{\varepsilon_0}{\mu_0} \right)^{\frac{1}{2}} k n_{\text{clad}}^2 \left\{ c_\nu^f \left[J_{\nu-1}(QR) + J_{\nu+1}(QR) \right] + c_\nu^s \left[H_{\nu-1}^{(1)}(QR) + H_{\nu+1}^{(1)}(QR) \right] \right\} \right.
$$

$$
\left. -\beta \left\{ d_\nu^f \left[J_{\nu-1}(QR) + J_{\nu+1}(QR) \right] + d_\nu^s \left[H_{\nu-1}^{(1)}(QR) + H_{\nu+1}^{(1)}(QR) \right] \right\} \right) \quad (12)
$$

where $U = r_{\text{co}}(k^2 n_{\text{co}}^2 - \beta^2)^{1/2}$, $V^2 = U^2 - Q^2$, and $R = r/r_{\text{co}}$.

Table 1. Coefficients appearing in Eqs. (1) and (2) for 'TM-like' (ITM) and 'TE-like' (ITE) radiation modes. These correspond to the pure TM and TE modes in the 'free-space' terms that are subsequently altered by the perturbation of the waveguide[1].

	ITE modes	ITM modes
a_ν	$-\frac{4}{\pi}\frac{\beta}{kn_{\rm co}^2}\frac{\nu}{kr_{\rm co}}\frac{V^2}{U^2Q^3}\frac{1}{J_\nu(U)H_\nu^{(1)}(Q)M_\nu}$	$-\frac{4}{\pi}\frac{n_{\rm cl}^2}{n_{\rm co}^2}\frac{1}{\beta r_{\rm co}Q}\frac{F_\nu}{J_\nu(U)H_\nu^{(1)}(Q)M_\nu}$
b_ν	$-\frac{4}{\pi}\left(\frac{\varepsilon_0}{\mu_0}\right)^{\frac{1}{2}}\frac{1}{Qkr_{\rm co}}\frac{G_\nu}{J_\nu(U)H_\nu^{(1)}(Q)M_\nu}$	$-\frac{4}{\pi}\frac{n_{\rm cl}^2}{n_{\rm co}^2}\left(\frac{\varepsilon_0}{\mu_0}\right)^{\frac{1}{2}}\frac{\nu}{kr_{\rm co}}\frac{V^2}{U^2Q^3}\frac{1}{J_\nu(U)H_\nu^{(1)}(Q)M_\nu}$
c_ν^f	0	$2i\frac{Q}{\beta r_{\rm co}}$
c_ν^s	$-\frac{4}{\pi}\frac{\beta}{kn_{\rm co}^2}\frac{\nu}{kr_{\rm co}}\frac{V^2}{U^2Q^3}\frac{1}{\left\{H_\nu^{(1)}(Q)\right\}^2M_\nu}$	$-2i\frac{Q}{\beta r_{\rm co}}\frac{J_\nu(Q)}{H_\nu^{(1)}(Q)}\frac{A_\nu}{M_\nu}$
d_ν^f	$2i\left(\frac{\varepsilon_0}{\mu_0}\right)^{\frac{1}{2}}\frac{Q}{r_{\rm co}k}$	0
d_ν^s	$-2i\left(\frac{\varepsilon_0}{\mu_0}\right)^{\frac{1}{2}}\frac{Q}{r_{\rm co}k}\frac{J_\nu(Q)}{H_\nu^{(1)}(Q)}\frac{B_\nu}{M_\nu}$	$-\frac{4}{\pi}\frac{n_{\rm cl}^2}{n_{\rm co}^2}\left(\frac{\varepsilon_0}{\mu_0}\right)^{\frac{1}{2}}\frac{\nu}{r_{\rm co}k}\frac{V^2}{U^2Q^3}\frac{1}{\left\{H_\nu^{(1)}(Q)\right\}^2M_\nu}$
N_j	$\frac{2\pi r_{\rm co}^2}{Q}\left(\frac{\varepsilon_0}{\mu_0}\right)^{\frac{1}{2}}\frac{\beta}{k}\times\begin{cases}1 & \text{for } \nu>0\\ 2 & \text{for } \nu=0\end{cases}$	$\frac{2\pi r_{\rm co}^2}{Q}\left(\frac{\varepsilon_0}{\mu_0}\right)^{\frac{1}{2}}\frac{kn_{\rm cl}^2}{\beta}\times\begin{cases}1 & \text{for } \nu>0\\ 2 & \text{for } \nu=0\end{cases}$

$$F_\nu = \frac{J_\nu'(U)}{UJ_\nu(U)} - \frac{H_\nu^{(1)'}(Q)}{QH_\nu^{(1)}(Q)} \tag{13}$$

$$G_\nu = \frac{J_\nu'(U)}{UJ_\nu(U)} - \frac{n_{\rm cl}^2}{n_{\rm co}^2}\frac{H_\nu^{(1)'}(Q)}{QH_\nu^{(1)}(Q)} \tag{14}$$

$$A_\nu = M_\nu - \frac{2i}{\pi}\frac{n_{\rm cl}^2}{n_{\rm co}^2}\frac{F_\nu}{Q^2J_\nu(Q)H_\nu^{(1)}(Q)} \tag{15}$$

$$B_\nu = M_\nu - \frac{2i}{\pi}\frac{G_\nu}{Q^2J_\nu(Q)H_\nu^{(1)}(Q)} \tag{16}$$

$$M_\nu = \left(\frac{\nu\beta}{kn_{\rm co}}\right)^2\left(\frac{V}{UQ}\right)^4 - F_\nu G_\nu \tag{17}$$

References

1. Snyder, A. W. & Love, J. *Optical Waveguide Theory* (Chapman and Hall Ltd, 1983), 1st edition edn.
